# Defining the Best Nasal Tip Projection among Iranian Women

**DOI:** 10.1155/2016/8549276

**Published:** 2016-05-16

**Authors:** Alireza Mohebbi, Hesam Jahandideh, Zhaleh Faham, Morteza Jafari

**Affiliations:** ^1^ENT-Head and Neck Surgery Department and Research Center, Hazrat Rasoul Akram Hospital, Iran University of Medical Sciences (IUMS), Tehran 1445613131, Iran; ^2^ENT-Head and Neck Surgery Department and Research Center, Firoozgar Hospital, Iran University of Medical Sciences (IUMS), Tehran, Iran

## Abstract

Rhinoplasty is one of the most complicated aesthetic surgeries. One important factor in nasal profile analysis before surgery is the NTP (Nasal Tip Projection). There has been controversy over defining the best tip projection and due to cultural differences there is a need to find the best formulation for Iranian noses. We selected 50 randomized patients. Lateral nasal views were captured from all of the patients. In order to equalize the photos, all tip rotations changed first to 105. We selected four methods for measuring NTP (Goode, Crumley 1, Crumley 2, and Powell and Humphreys). Based on these methods NTP was shown in four pictures. A questionnaire was designed for rating the pictures. Questionnaires were filled in by 3 different groups: rhinoplasty surgeons, general people, and artists. A total of 73 questionnaires were filled in. The analysis and comparison were done. Crumley 2 is the best NTP measurement method from the surgeons' and artists' view. Goode is the method preferred by general people. Powell & Humphreys method seems to be the worst method from all 3 groups' view. It seems that general people prefer smaller noses, because projection in Goode method is almost less than Crumley 2.

## 1. Introduction

Rhinoplasty is one of the most complicated aesthetic surgeries. As a result of central anatomic place of the nose in the face it has a great importance in overall beauty. One of the important factors in nasal profile analysis to be considered before surgery is the NTP (Nasal Tip Projection). Hence no standard is yet accepted for ideal NTP; facial aesthetic analysis is one of the important and basic issues to define before the surgery. The literature divides patients after surgery to 3 groups: first group includes satisfied patients and surgeons, 2nd one which is the most prevalent one is the group where patients are satisfied while the surgeons are not, and the last group includes both unsatisfied surgeons and unsatisfied patients [1]. NTP and nasal tip rotation are related strongly. Nasofrontal Angle (NFA) has less importance compared to Nasofacial and Nasolabial Angles (NLA) [2]. Toriumi announced that nose should be in a shape that can make eyes and other important parts become bolded in the face [3].

Facial anthropometry among different nations is different and one of the most important factors regarding this issue is nasal shape [4–6]. There has been controversy over defining the best tip projection and due to cultural differences, there is a need to find the best formulation and method for Iranian noses plastic surgery. There are different methods to formulate NTP. According to Baum definition, for the best NTP, the ratio between the line from TDP (Tip Defining Point) perpendicular to the line between NFA and NLA must be 1 : 2 [7]. In a similar way Powell and Humphreys used the same perpendicular line but the ratio must be 1 : 2.8 [8]. Simons used the upper lip length for calculating the best NTP; this usage makes this method different from the others. By Simons definition the upper lip length must be the same as base of nose's length [9]. Goode method uses a right triangle to define best NTP. The first side of the triangle is a line from NFA to Alar Crease, the second side is a perpendicular line from TDP to this line, and the third line is on the nasal dorsum [9]. Crumley and Lanser recommends 2 new ways for formulating best NTP. In Crumley 1 the ratio between the line from nasion to the vermillion and upper lip's skin junction perpendicular to the line from TDP must be 3.53. Crumley 2 uses a triangle just like Goode but the posterior line continues its way and reaches the mandible profile at last, the ratio between this line with the perpendicular line to it must be 4.23 [10]. The literature announces Goode and Crumley as better methods for the facial attraction [11]. As a result we chose the most preferred methods to formulate best NTP among Iranian women: Crumley 1 and Crumley 2, Powell and Humphreys, and Goode methods.

As mentioned previously, no standard is yet accepted for ideal NTP; also there has been controversy over defining the best tip projection and considering the cultural differences around the world; we tried to find the relationship between facial attractiveness and NTP measurement methods among Iranian females by comparing ideals between nose surgeons, general people, and artists (portrait sketchers). Beside what has been noted recently, we have to consider rhinoplasty as a prevalent surgery among Iranian women; as a result we need to define ideal NTP among Iranian women.

## 2. Methods

We selected 50 patients' lateral nose view randomly from a total of 1000 patients who attend a private office. The excluding criteria were suffering from a craniofacial deformity, having a considering nose's hump, and whether the patient did not consent to participate in our study. The patients' age was from 20 to 40 years.

**Figure 1:**
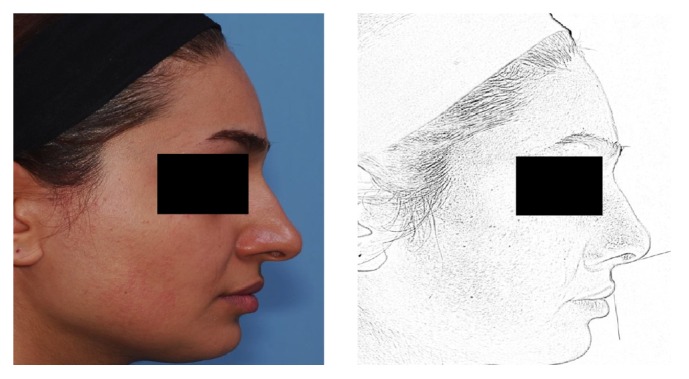


Lateral nasal views were captured. In order to equalize the photos, all tip rotations changed to 105 (women normal range: 95–110). As noted we selected four methods for measuring NTP (Goode, Crumley 1, Crumley 2, Powell and Humphreys); based on them NTP was shown in four different pictures (A, B, C, and D, resp.) which were placed near each other for making judgment easier. We worked on all pictures with Photoshop software (version CS5) and did our best to save the other specialties of the photos such as light and color being the same as each other (Figure 1).

We asked the participants to rate each of the four pictures of all 50 patients. The best one was rated as number 1 and others were rated as 2, 3, and 4, respectively. A questionnaire was designed to rate the pictures. Questioning was done among 3 groups: rhinoplasty surgeons (12 people), general people (46 persons), and portrait painters as artists (13 persons).

General peoples were selected among relatives of patients who were admitted in hospital because of noncosmetic surgeries.

The gathered data was analyzed with SPSS version 13. The mean mark in each group was calculated and compared between the groups.

## 3. Results

A total of 73 questionnaires were filled in. 2 of them were not completed. From a total 71 completely filled questionnaires 16.9% were filled by surgeons, 64.8% were filled in by general people, and 18.3% were filled in by artists. Mean score of each picture is shown in Table 1.

Among surgeons the least score was for picture 3 (C) which was 1.87 and it enhances.

Crumley 2 is the best method from surgeons' view. General people gave the best mark 2.12 to picture 1 (Goode method), but artists just like surgeons chose picture 3 as the best one with mean mark of 2.10.

By ANOVA scale, for picture A there was no significant different in mean scores among 3 groups (*P* value = 0.37). In the other three pictures there were significant differences found in mean scores (*P* value < 0.05). In picture B surgeons' idea was completely different from other groups (*P* value = 0.00). In pictures D and C surgeons idea was significantly different from general people but not from artists. (*P* value = 0.002 and 0.011, resp.).

Among surgeons and artists, picture C was the favorite; about 41.5% of all surgeons and 34% of artists gave 1 as these pictures scores. As said before 1 was the best mark in our method. Among general people picture A was the favorite and 40% of participants gave 1 to this picture.

## 4. Discussion

Crumley 2 is the best NTP measurement method by the surgeons and artists' taste in Iran. Goode is the best method from general people's view. It seems that general people prefer smaller noses, because projection in Goode method is almost less than Crumley 2. Our results indicate that rhinoplasty surgeons' opinions are similar to portrait artists'.

The satisfaction after Rhinoplasty is affected by different factors. One of these factors is NTP. Although NTP is a very important factor there has not been a standard for NTP measurement yet. Furthermore facial anthropometry is not the same among different nations [4–6]. Among eastern Asian nations the most prevalent problem is lack of septal cartilage which is needed for sufficient nose projection; there are some different methods for their surgeons such as flag technique. But among Iranian people the most common anomaly is alar cartilage malposition (51%) and others are low radix (36%), inadequate tip projection (35%), and middle vault collapse (15%). Frequency of low radix in male patients is 2.5 times more than females. We have to consider that we cannot use other nations' NTP profile for Iranian people. Inadequate tip projection which is prevalent among Iranian women highlights the need of having a specified method for analyzing the best tip projection among them. We found that among general Iranian women Goode is the most pleasant method and Crumley is the pleasant one among surgeons and artists. Devcic et al. supporting our data announce that Crumley and Goode are the best methods for making the patients facial abilities become more attractive [11–13].

Powell and Humphreys was the least pleasant method among all three groups. But we should not forget that the marks among each group varied widely which shows us that the better NTP is related to the taste of participants even among surgeons.

One limitation of our study was the large amount of photos (200) which became time-consuming for our participants, mostly for surgeons and artists, which made us have some incomplete questionnaires for excluding. One other limitation of our study is that we only studied NTP but do not work on nose beauty after surgery from other sides such as front view or oblique one.

To conclude, Crumley 2 is the best NTP measurement method chosen by the surgeons and artists. Goode is the best method among general people's taste. No standard is yet accepted for ideal NTP. As patients' satisfaction from the nose job results from their imagination about an ideal nose shape before surgery, it seems that we need a 3D method to formulate nasal dimensions alongside other facial subunits.

## Figures and Tables

**Table 1:** 

	Responder					
	Surgeon		General people		Artist	
	Mean	SD^*∗*^	Mean	SD	Mean	SD
Picture 1 (A)	2.10	0.50	2.12	0.43	2.30	0.28
Picture 2 (B)	1.97	0.22	2.47	0.44	2.48	0.17
Picture 3 (C)	1.87	0.24	2.27	0.39	2.10	0.25
Picture 4 (D)	2.70	0.42	3.12	0.45	3.12	0.34

SD^*∗*^ Standard deviation.
